# The Financial Burden of Morbidity in HIV-Infected Adults on Antiretroviral Therapy in Côte d'Ivoire

**DOI:** 10.1371/journal.pone.0011213

**Published:** 2010-06-18

**Authors:** Arnousse Beaulière, Siaka Touré, Pierre-Kébreau Alexandre, Koko Koné, Alex Pouhé, Bertin Kouadio, Neige Journy, Jérôme Son, Virginie Ettiègne-Traoré, François Dabis, Serge Eholié, Xavier Anglaret

**Affiliations:** 1 INSERM, Unité 897, Université Victor Segalen Bordeaux 2, Bordeaux, France; 2 Programme PAC-CI, Abidjan, Côte d'Ivoire; 3 Department of Mental Health, Bloomberg School of Public Health, Johns Hopkins University, Baltimore, Maryland, United States of America; 4 Association ACONDA-VS, Abidjan, Côte d'Ivoire; 5 Institut Pédagogique National de l'Enseignement Technique et Professionnelle (IPNETP), Abidjan, Côte d'Ivoire; 6 Programme National de Prise En Charge des Personnes infectées par le VIH (PNPEC), Ministère de la Santé et de l'Hygiène Publique, Abidjan, Côte d'Ivoire; 7 Service des Maladies Infectieuses et Tropicales, Centre Hospitalier Universitaire (CHU) de Treichville, Abidjan, Côte d'Ivoire; Erasmus University Rotterdam, Netherlands

## Abstract

**Background:**

Large HIV care programs frequently subsidize antiretroviral (ARV) drugs and CD4 tests, but patients must often pay for other health-related drugs and services. We estimated the financial burden of health care for households with HIV-infected adults taking antiretroviral therapy (ART) in Côte d'Ivoire.

**Methodology/Principal Findings:**

We conducted a cross-sectional survey. After obtaining informed consent, we interviewed HIV-infected adults taking ART who had consecutively attended one of 18 HIV care facilities in Abidjan. We collected information on socioeconomic and medical characteristics. The main economic indicators were household capacity-to-pay (overall expenses *minus* food expenses), and health care expenditures. The primary outcome was the percentage of households confronted with catastrophic health expenditures (health expenditures were defined as catastrophic if they were greater than or equal to 40% of the capacity-to-pay). We recruited 1,190 adults. Median CD4 count was 187/mm^3^, median time on ART was 14 months, and 72% of subjects were women. Mean household capacity-to-pay was $213.7/month, mean health expenditures were $24.3/month, and 12.3% of households faced catastrophic health expenditures. Of the health expenditures, 75.3% were for the study subject (ARV drugs and CD4 tests, 24.6%; morbidity events diagnosis and treatment, 50.1%; transportation to HIV care centres, 25.3%) and 24.7% were for other household members. When we stratified by most recent CD4 count, morbidity events related expenses were significantly lower when subjects had higher CD4 counts.

**Conclusions/Significance:**

Many households in Côte d'Ivoire face catastrophic health expenditures that are not attributable to ARV drugs or routine follow-up tests. Innovative schemes should be developed to help HIV-infected patients on ART face the cost of morbidity events.

## Introduction

The World Health Organization (WHO) estimated that 480,000 adults and children were living with HIV in Côte d'Ivoire, West Africa in late 2007. Of the 190,000 persons who were estimated to be in need of antiretroviral therapy (ART), 51,812 (28%) were actually receiving ART [1]. Most of these patients had started ART within the previous three years, primarily thanks to support from the US President's Emergency Plan for AIDS Relief (PEPFAR) and the Global Fund to Fight AIDS, Tuberculosis and Malaria [2].

In most African countries that receive funds from these international programs, antiretroviral drugs and follow-up tests have become widely available at relatively low costs. In Côte d'Ivoire, HIV-infected patients paid a fixed rate of US$1.5 per month per family from 2005 to 2008, to have access to ARV drugs and CD4 count tests [3]. As of August 2008, ARV drugs and CD4 tests are entirely free for patients. All other health care expenses, including cotrimoxazole prophylaxis, diagnostic tests, and treatment of morbidity events after ART initiation, however, continue to be borne by the patients [1], [2]. The HIV virus reduces the concentration of CD4+ T cells in the blood, thus suppressing the immune system and increasing the risk of developing opportunistic diseases. As CD4 counts decrease, the incidence of opportunistic diseases increases. ART curbs viral replication in patients infected with HIV, thereby restoring the immune system, increasing the CD4+ T cell count, and reducing the risk of morbidity.

The financial burden of intercurrent morbidity events for HIV-infected patients must be determined as a function of CD4 count, because it is likely to decline rapidly after ART initiation and increases in CD4 counts [4]–[6]. Although it is reasonable to expect that the financial burden of HIV on households decreases considerably after ART initiation, preliminary evidence suggests the contrary [2], [4]–[6]. To explore this issue, the Côte d'Ivoire Ministry of Health has recommended that studies be conducted on health care expenses among HIV-infected patients initiating ART in Côte d'Ivoire.

Here we report the health-related expenditures of adults on ART in Côte d'Ivoire.

## Methods

### Data collection

We conducted a two-month cross-sectional survey from June to July 2007 in 18 health care facilities, 16 in Abidjan, the economic capital, and two in provincial towns. These public or non-profit private health centres all participate in the Aconda HIV care network. Aconda, a non-governmental organization that is dedicated to HIV/AIDS care and treatment, has been described elsewhere [7]. Study participants were selected sequentially. One patient out of every X patients who presented to care between March 1 and April 30, 2007 was asked to participate. The inclusion rate X differed by study center and was equal to A/(B×70%), where A was the sample size at the study center, B was the anticipated number of patients who would visit the center during the study period, and 70% was the anticipated proportion of patients who would accept to participate. The sample size A at each study center was the overall study sample size multiplied by the number of patients in active follow-up at the study center and divided by the number of patients in active follow-up at all 18 study centers.

Overall, 1,451 adults taking ART who had attended one of the 18 HIV care centres were randomly chosen to participate in a face-to-face interview. 1,275 HIV-infected patients accepted to be interviewed and gave verbal informed consent – informed consent took the form of an open, easily understood communication process between investigator and subject approved by the national ethics committee. After obtaining verbal informed consent, investigators used a standardized questionnaire to record data on household structure, and expenses during the past three months.

When patients did not have some of the requested information, we asked for their permission to contact the head of the household or any other adult household member who could answer our questions. The unit of observation was one patient per household.

Individual data on HIV disease, including most recent CD4 count and history of ART, were gathered from each patient's personal medical record at the HIV care centre.

This study was approved by the Ethics Committee of the Côte d'Ivoire Ministry of Health and the Institutional Review Board of the French National Agency for AIDS research and viral hepatitis (ANRS).

### Variables definition

Total household expenditures were defined as the sum of the expenses of all household members over the past three months. Expenditures were classified as food expenditures, health care expenditures, and other expenditures (electricity, water, telephone, school fees, transportation non-related to health, etc) [8], [9].

Household capacity-to-pay was defined as the household's non-subsistence expenses, i.e. total household expenditures *minus* food expenditures [10]–[15].

Health expenditures were stratified into two groups: expenditures related to treatment of the HIV-infected study subject, and expenditures related to the treatment of other members of the family (whose HIV serostatus was unknown). Health expenditures for the HIV-infected study subjects were divided into three groups: (i) ARV drugs and routine follow-up tests, which were available to all HIV-infected patients living in Côte d'Ivoire at a fixed rate of US$ 1.5 per month at the time of the survey; (ii) other medical expenditures (medical consultations, hospital stays, non-CD4 tests, and non-ARV drugs), and (iii) non-medical health-related expenditures (transportation to HIV care centres and housing near health facilities).

Health expenditures are defined as catastrophic when they exceed a given proportion of household resources [13], [16]. We used the WHO definition, which characterizes health expenses as catastrophic when they are greater than or equal to 40% of a household's capacity-to-pay [11].

### Statistical analysis

Expenses were estimated in US dollars ($). Each variable was estimated per household. We determined the mean, standard deviation, median, interquartile range, and range of the distribution of each household characteristic in the study population. Data were described both overall, and stratified by most recent CD4 count (<100, 100–199, 200–349, 350–499, and ≥500/mm^3^). We analysed differences in main outcomes by CD4 category using parametric or non-parametric tests, depending on the distribution of the variable. We determined the factors associated with catastrophic health expenditures using univariate and multivariate logistic regression.

Analyses were performed using STATA 10 software (StataCorp, College Station, Texas, USA).

## Results

### Socio-demographic and clinical characteristics

Among the 1,275 HIV-infected patients on ART who accepted to be interviewed and gave verbal informed consent, 85 (6.7%) were excluded from the analysis, because their CD4 counts had not been measured in the previous 12 months. Of the remaining 1,190 patients that we included in the analysis, 72% were female and 45% were heads of household. The main clinical and socio-demographic characteristics of these patients are reported in Table 1. Nine percent of households had a health insurance scheme.

**Table 1:** Characteristics of patients and households (N = 1190).

Patient characteristics	
Most recent CD4 count (cell/mm^3^), median (IQR)	187 (88–301)
Median time (months) since most recent CD4 count, median (IQR)	5.5 (3.4–7.5)
CD4 stratum (cell/mm^3^), number (%)	
<100	330 (27.7)
100–199	301 (25.3)
200–349	332 (27.9)
350–499	124 (10.4)
≥500	104 (8.7)
Time spent on ART (months), median (IQR)	14 (7–23)
Age (years), median (IQR)	37 (31–43)
Women, number (%)	857 (72)
Employment sector of the patient, number (%)	
Formal	167 (14)
Informal	559 (47)
Unemployed	464 (39)
Patient is household head, number (%)	535 (45)

Structure of the households	
Number of household members, median (IQR)	5 (3–8)
Level of education of the household head, number (%)	
None	500 (42)
Primary	286 (24)
Secondary	309 (26)
University	95 (8)

**Footnotes for table 1:**

%: percentage.

IQR: interquartile range.

ART: antiretroviral therapy.

Most recent CD4 count: all within the past 12 months; median time since the most recent CD4 count was 5.5 months (IQR 3.4; 7.5).

Formal sector: public or private sector employment registered with the income (or profit) tax authorities.

Informal sector: employment in at least one unorganized enterprise in the last 7 days, irrespective of employment status and regardless of whether it was the primary or secondary job.

### Expenditures

Investigators collected information from 1,158 patients (97.3%) on household structure and expenses in order to complete the questionnaire. We interviewed other adult household members to complete the questionnaire for the remaining 32 (2.7%) patients.

Mean overall household expenditures were $313.8 per household per month (Table 2). When we subtracted food from the overall household expenditures, mean household capacity-to-pay was $213.7 per month. Health care expenditures accounted on average for 9.9% of overall household expenditures, and 17.7% of the capacity-to-pay (standard deviation 0.5%, median 10.4%, interquartile range [IQR] 5.2%–21.9%). On average, 75.3% of health expenditures were for the study subjects (ARV drugs and CD4 tests, 24.6%; other tests and drugs, 50.1%; and transportation to HIV care centre, 25.3%), and 24.7% were for other members of the household.

**Table 2:** Household expenditures.

	Mean value US$ per month (SD)	Mean percentage		
Total household expenditures*	313.8 (14.9)	**100%**		
Food expenditures	100.1 (2.2)	39.7%		
Non-food non-health expenditures	189.4 (14.2)	50.5%		
Health expenditures	24.3 (1.5)	9.9%	**100%**	
For the HIV-infected study subject	13.8 (0.7)	-	75.3%	**100%**
Antiretroviral drugs and CD4 count tests	1.9 (0.0)	-	-	24.6%
Other medical costs**	8.8 (0.7)	-	-	50.1%
Transportation	3.1 (0.2)	-	-	25.3%
Other members of the household	10.5 (1.2)	-	24.7%	-

**Footnotes for table 2:**

ARV: antiretroviral.

Capacity-to-pay: non-subsistence expenses, i.e. total household expenditures *minus* food expenditures.

*Self-declared expenditures in FCFA converted at exchange rate US$1 = 420 FCFA at time of survey (June–July 2007).

**Other drugs and tests, medical consultations, and hospital stays.

**Figure 1:**
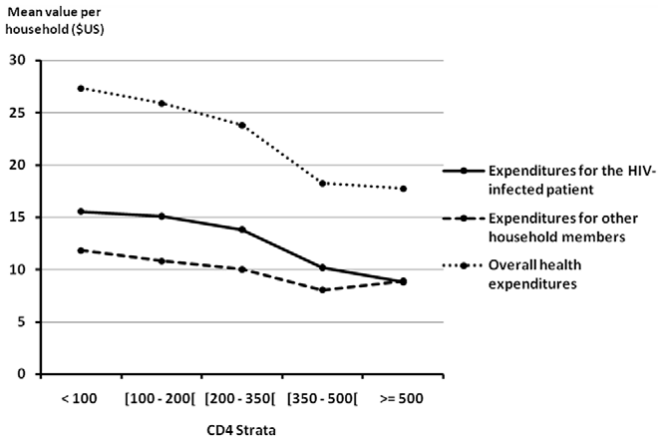
Household health expenditures for HIV-infected patients and other members of the family by CD4 count. **Footnotes for figure 1:** • Expenditures for the HIV-infected patient by CD4 count, overall *p* value<0.001. • Expenditures for other household members by CD4 count, overall *p* value = 0.77. • Overall health expenditures by CD4 count, overall *p* value = 0.03.

**Figure 2:**
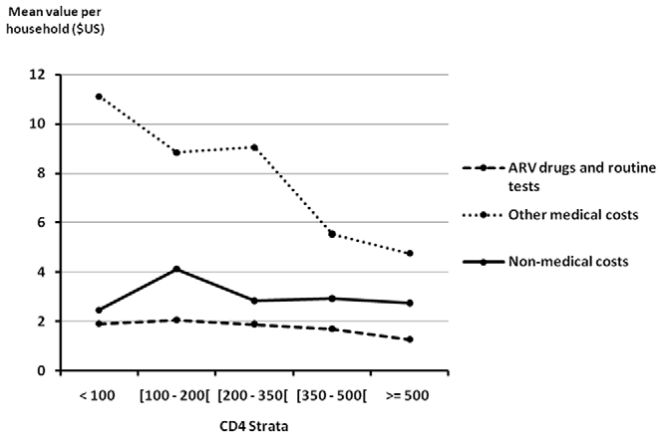
Categories of health expenditures for HIV-infected patients by CD4 count. **Footnotes for figure 2:** • ARV drugs and routine tests by CD4 count, overall *p* value<0.001. • Other medical costs (non ARV drugs, non-routine tests, consultations, hospital stays) by CD4 count, overall *p* value = 0.0001. • Non-medical costs (transportation) by CD4 count, overall *p* value = 0.14.

Figure 1 and Figure 2 illustrate the components of household health expenditures, stratified by most recent CD4 count. As shown in Figure 1, overall health expenditures and expenditures related to treatment of the study subject were significantly lower when patients had higher CD4 counts (p = 0.03 and p<0.001, respectively). Health expenditures for other members of the household did not differ significantly by CD4 count (p = 0.77). As shown in Figure 2, spending on ARV drugs and routine tests was slightly lower, and spending on non-ARV drugs and non-CD4 tests was dramatically lower, when patients had higher CD4 counts. Both differences were significant (p<0.001 and p<0.001, respectively).

**Figure 3:**
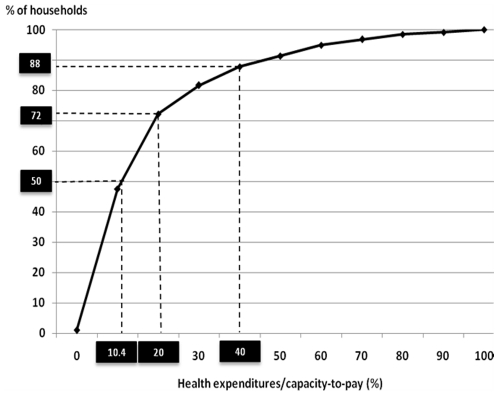
Health expenditures/capacity-to-pay ratio.

Figure 3 shows the percentage of households whose health expenditures/capacity-to-pay ratios reached various thresholds. Twelve percent of households met the WHO definition for catastrophic health care expenditures (health expenditures greater than or equal to 40% of the household's capacity-to-pay), 28% of households had ratios above 20%, and 50% of households had ratios above 10.4%.

The following factors were associated with catastrophic health expenditures in multivariate analysis (Table 3): increased time spent on ART (adjusted odds ratio [AOR] 0.97; 95% confidence interval [CI] 0.94–0.99), female HIV-infected patients (AOR 0.58; 95%CI 0.46–1.33), secondary level of education of the household head (AOR 0.31; 95%CI 0.15–0.63), increased household size (AOR 0.73; 95%CI 0.66–0.81); highest household income quartile (AOR 0.27; 95%CI 0.07–1.12). There was no significant association between catastrophic health expenditures and household having a health insurance scheme (AOR 0.59; 95%CI 0.13–2.55).

**Table 3:** Factors associated with catastrophic health expenditures (threshold/cut-off level = 40%) among household with HIV-infected patients.

	AOR (95%CI)	P-value
**Patient characteristics**		
CD4 (cells/mm^3^)		
≤200	0.87 (0.45–1.67)	0.68
[200–350]	1.04 (0.51–2.11)	0.91
≥350	1	
Time spent on ART	0.97 (0.94–0.99)	0.00
Age (years)	1.02 (0.99–1.05)	0.19
Patient is household head		
Yes	1	
No	0.79 (0.46–1.35)	0.38
Female		
Yes	1	
No	0.58 (0.46–1.33)	0.04
Employment sector of the patient		
Formal	1	
Informal	1.80 (0.69–4.66)	0.23
Unemployed	1.90 (0.41–8.79)	0.41
**Household characteristics**		
Income quartiles		
Lowest quartile	1	
Lower middle quartile	2.45 (0.65–9.17)	0.18
Upper middle quartile	1.13 (0.33–3.85)	0.85
Highest quartile	0.27 (0.07–1.12)	0.07
Household size	0.73 (0.66–0.81)	0.00
Insurance status		
Yes	1	
No	0.59 (0.13–2.55)	0.48
Level of education of the household head		
No education	1	
Primary	0.88 (0.53–1.44)	0.61
Secondary	0.31 (0.15–0.63)	0.00
University	-	-

**Footnotes for table 3:**

AOR: Adjusted Odds Ratio; CI: Confidence Interval.

ART: antiretroviral therapy.

Most recent CD4 count: all within the past 12 months; median time since the most recent CD4 count was 5.5 months (IQR 3.4; 7.5).

Formal sector: public or private sector employment registered with the income (or profit) tax authorities.

Informal sector: employment in at least one unorganized enterprise in the last 7 days, irrespective of employment status and regardless of whether it was the primary or secondary job.

Differences between patients who were excluded due to missing CD4 counts and patients who were included in the study were significant for mean age (34 *vs.* 37 years, p<0.01) and mean health expenditures among HIV-uninfected members of households ($5.8 *vs.* $10.4/month, p = 0.01). Differences in all other variables, including total household expenditures, total health expenditures and food expenditures, were not significant.

## Discussion

We investigated the burden of health care expenditures among households with HIV-infected adults on ART in Côte d'Ivoire, West Africa. Our findings add to the literature on the economic impact of chronic diseases such as HIV/AIDS on households in resource-limited settings [6], [12]–[20].

In the early era of large HIV care and treatment programmes in sub-Saharan Africa, the emphasis has been on improving access to ARV drugs and routine tests. Previous studies have shown that out-of-pocket payments for drugs and tests act as a barrier to care, and that clinical outcomes are worse in settings in which drugs and routine tests are not free [4], [21], [22], [23], [24]. These findings have largely contributed to the decision of program funders to make ARV drugs and routine tests available free of charge throughout the continent.

ARV drugs and routine tests were available at a very low cost in Côte d'Ivoire at the time of our study, and have recently become entirely free. Our study suggests that this decision is unlikely to alleviate the economic burden of HIV care on households, because the majority of health expenditures are for non-ARV drugs, non-routine tests, medical consultations and hospital stays. A significant proportion of households with patients on ART, therefore, continue to be confronted with catastrophic health expenditures. Catastrophic health expenditures are defined as health care costs that constitute a large share of household resources and either divert from consumption of basic goods or force the household to use savings, sell assets, or borrow money [10], [25]. Our results strongly suggest that morbidity events in patients on ART represent a significant economic burden for households, even when patients have CD4 counts above 200 [26]. In multivariate analysis, the risk of catastrophic household health expenditures decreased with increased time spent on ART, female HIV-infected patients, head of household education level and household size. As expected, households with higher incomes were at lower risk of incurring catastrophic health expenditures than households with lower incomes [14], [27]. Several hypotheses may explain our finding that the households of HIV-infected women are less likely to be associated with catastrophic spending. First, female patients may have unequal access to care compared to men. Their health expenditures may therefore be lower than those of male patients. Our finding is more likely due, however, to the fact that women in Côte d'Ivoire are generally not the main income earner of their household. HIV-infected women thus have a smaller impact on their households than HIV-infected men. When the HIV-infected person is the main income earner, time since infection becomes a critical factor in determining whether household expenditures will become catastrophic. If the patient's disease worsens, health expenditures increase and other household expenditures become more difficult to sustain. We also found that the presence or absence of health insurance had no impact on results, suggesting that having insurance does not protect households from catastrophic spending.

Our study has several limitations. First, we only calculated direct costs. If we had integrated indirect costs, such as time and opportunity costs, we likely would have found that morbidity events in patients on ART with low or intermediate CD4 counts leads to losses in income and productivity [12], [17], [28]. Income losses reduce household capacity-to-pay, while productivity losses result in a burden for the community. We probably took some income losses into account when we estimated overall household expenses and capacity-to-pay, but our survey did not deal with productivity losses. Therefore, we cannot determine the impact of non-ART and non-routine HIV care on the community.

Second, we estimated the household burden of health expenditures by estimating the proportion of the capacity-to–pay that was spent on health care. Capacity-to-pay was estimated from overall household expenditures, not household self-declared income. We used this method, because the sum of self-reported expenditures is considered to be a better approximation of the household's real purchasing power than the sum of self-reported incomes [9]. However, this method does not consider health expenditures that may have been reimbursed by insurance companies. We may have overestimated the burden of health expenditures among the 9% of households who declared having health insurance [8]. However, households with health insurance are generally wealthier than those without it [29], so it is unlikely that they introduced a major bias in our main results.

This study likely underestimated the economic consequences of HIV care on households, for several reasons. First, we were only able to gather data on one HIV-infected patient per household. In households with more than one HIV-patient, total household health expenditures were probably significantly higher. Second, we excluded 6.7% of patients from the study because they had not performed CD4 count tests. Although total household expenditures and total health expenditures did not differ significantly between excluded patients and included patients, it is still possible that excluded patients were poorer than patients who were included in the study. Third, some authors suggest using shorter recall periods for regular purchases like food, and longer recall periods for durable goods such as rent [15]. Because we asked households to recall all expenditures, including food and housing, in the previous 3 months, we may have underestimated both regular purchases and durable goods. Fourth, 16 of the 18 study centers were in the economic capital of Côte d'Ivoire and probably provide care to the wealthier segments of the country's population. Catastrophic health expenditures are likely to be even higher in other regions of Côte d'Ivoire. Finally, some households may have been using outside funds to cope with the health expenditures associated with HIV at the time of the survey, as suggested by previous studies [12], [18], [30]. The use of precautionary savings, depletion of assets, and money borrowed from friends and relatives to pay for both health care and other expenses (e.g. school) may have inflated the capacity-to-pay and reduced the perceived proportion of households with catastrophic health expenditures [12], [13], [15], [30]–[33].

To our knowledge, no other study in Côte d'Ivoire has estimated the expenditures of households with at least one HIV-infected member. We therefore cannot compare our results to previous studies. Nor can we declare that our sample is representative, because our survey was not conducted at the national level. That said, a 2008 national survey of the standard of living in households across Côte d'Ivoire (National Household Living Standard Survey) found that 47.8% of household expenses were related to food, 5.8% were related to health care and 3.7% were related to education [34]. In our study, the proportion of total household expenditures devoted to food (39.7%) was lower than in the general population, while the proportion of total household expenditures used for health-related needs (9.9%) was much higher than in the general population.

Finally, the time since HIV diagnosis at the time of the survey was unknown. It is likely that as households continue to cope with HIV-related health expenses over time, the financial burden becomes increasingly large.

We used a 40% health expenditures/capacity-to-pay ratio to define catastrophic health expenditures, consistent with the WHO definition [10], [14], [25]. Other studies have used thresholds of 5% or 20% to define health expenditures as high burdens for the household [12], [27], [35]. When we varied the threshold from 20% to 60%, the percentage of households facing catastrophic health expenditures was 10–28%. While the 40% ratio is commonly used, there is no definitive threshold for establishing when expenditures become catastrophic [14]. This approximate threshold represents the limit under which households must sacrifice basic needs, sell productive assets, incur debt, or be impoverished, in order to finance health expenditures [12], [30], [36].

In conclusion, our findings suggest that innovative schemes should be developed to help HIV-infected patients on ART face the cost of morbidity events. The recent decision by the government of Côte d'Ivoire to provide ARV drugs and CD4 tests for free should be considered as a first step, but additional steps must be taken to further alleviate the burden of HIV/AIDS care and treatment in patients on ART [37]–[40].

Abolishing user fees would mean higher real costs for funders [41], [42]. Financing strategies, such as pre-payments and community-based or micro-insurance risk pooling, could be implemented to make households participate in reducing costs, while simultaneously preventing catastrophic financial situations. The aim of a new financing system should be to improve access to care, especially for concurrent morbidity events, to protect people from catastrophic financial expenses and subsequent impoverishment, and to offer equitable and efficient health benefits [43]–[48]. One attractive operational scheme might be a National HIV/AIDS Care Equity Fund, which could be financed by international donors, national authorities and households. This type of system, however, should not be designed in isolation, but in combination with strategies, including a national social security system and public-private partnerships [49]–[51]. Further studies should assess the feasibility, sustainability and operational challenges of a National HIV/AIDS Care Equity Fund.

## References

[1] WHO (2008). The World Health Report 2008. Primary health care. Now more than ever.

[2] WHOUNAIDSUnicef (2009). Toward universal access. Scaling up priority HIV/AIDS interventions in the health sector. Progress report 2009.

[3] Ministère de la Lutte contre le Sida (2008). Suivi de la déclaration d'engagement sur le VIH/SIDA (UNGASS). Rapport national de la Côte d'Ivoire 2008.

[4] Brinkhof MW, Boulle A, Weigel R, Messou E, Mathers C (2009). Mortality of HIV-infected patients starting antiretroviral therapy in sub-Saharan Africa: comparison with HIV-unrelated mortality.. PLoS Med.

[5] Boyer S, Marcellin F, Ongolo-Zogo P, Abega SC, Nantchouang R (2009). Financial barriers to HIV treatment in Yaounde, Cameroon: first results of a national cross-sectional survey.. Bull World Health Organ.

[6] Onwujekwe O, Dike N, Chukwuka C, Uzochukwu B, Onyedum C (2009). Examining catastrophic costs and benefit incidence of subsidized antiretroviral treatment (ART) programme in south-east Nigeria.. Health Policy.

[7] Toure S, Kouadio B, Seyler C, Traore M, Dakoury-Dogbo N (2008). Rapid scaling-up of antiretroviral therapy in 10,000 adults in Cote d'Ivoire: 2-year outcomes and determinants.. AIDS.

[8] Murray CJ, Evans DB (2003). Health systems performance assessment: debates, methods and empiricism.

[9] Deaton A (1997). The analysis of household surveys: a microeconometric approach to development policy.

[10] Xu K, Evans DB, Kawabata K, Zeramdini R, Klavus J (2003). Household catastrophic health expenditure: a multicountry analysis.. Lancet.

[11] Xu K, Evans DB, Carrin G, Aguilar-Rivera AM (2005). Designing health financing systems to reduce catastrophic health expenditure. Technical briefs for policy-makers no 2.

[12] Russell S (2004). The economic burden of illness for households in developing countries: a review of studies focusing on malaria, tuberculosis, and human immunodeficiency virus/acquired immunodeficiency syndrome.. Am J Trop Med Hyg.

[13] Wagstaff A, van Doorslaer E (2003). Catastrophe and impoverishment in paying for health care: with applications to Vietnam 1993–1998.. Health Econ.

[14] van Doorslaer E, O'Donnell O, Rannan-Eliya RP, Somanathan A, Adhikari SR (2007). Catastrophic payments for health care in Asia.. Health Econ.

[15] Somkotra T, Lagrada LP (2009). Which households are at risk of catastrophic health spending: experience in Thailand after universal coverage.. Health Aff (Millwood).

[16] Wyszewianski L (1986). Families with catastrophic health care expenditures.. Health Serv Res.

[17] Veenstra N, Whiteside A (2005). Economic impact of HIV.. Best Pract Res Clin Obstet Gynaecol.

[18] McIntyre D, Thiede M, Dahlgren G, Whitehead M (2006). What are the economic consequences for households of illness and of paying for health care in low- and middle-income country contexts?. Soc Sci Med.

[19] Hounton SH, Akonde A, Zannou DM, Bashi J, Meda N (2008). Costing universal access of highly active antiretroviral therapy in Benin.. AIDS Care.

[20] Taverne B, Diop K, Vinard P, Coriat B (2008). The cost of universal free access for treating HIV/AIDS in low-income countries: the case of Senegal.. The political Economy of HIV/AIDS in Developing Countries TRIPS, Public Health Systems and Free Access.

[21] Msellati P, Juillet-Amari A, Prudhomme J, Akribi HA, Coulibaly-Traore D (2003). Socio-economic and health characteristics of HIV-infected patients seeking care in relation to access to the Drug Access Initiative and to antiretroviral treatment in Cote d'Ivoire.. AIDS.

[22] Katzenstein D, Laga M, Moatti JP (2003). The evaluation of the HIV/AIDS drug access initiatives in Cote d'Ivoire, Senegal and Uganda: how access to antiretroviral treatment can become feasible in Africa.. AIDS.

[23] Furber AS, Hodgson IJ, Desclaux A, Mukasa DS (2004). Barriers to better care for people with AIDS in developing countries.. BMJ.

[24] Posse M, Meheus F, van Asten H, van der Ven A, Baltussen R (2008). Barriers to access to antiretroviral treatment in developing countries: a review.. Trop Med Int Health.

[25] Xu K, Evans DB, Kadama P, Nabyonga J, Ogwal PO (2006). Understanding the impact of eliminating user fees: utilization and catastrophic health expenditures in Uganda.. Soc Sci Med.

[26] Nombela N, Kouadio B, Toure S, Seyler C, Flori YA (2006). Nonantiretroviral drug consumption by CD4 cell count in HIV-infected adults: a 5-year cohort study in Cote d'Ivoire.. J Acquir Immune Defic Syndr.

[27] Su TT, Kouyate B, Flessa S (2006). Catastrophic household expenditure for health care in a low-income society: a study from Nouna District, Burkina Faso.. Bull World Health Organ.

[28] Duraisamy P, Ganesh AK, Homan R, Kumarasamy N, Castle C (2006). Costs and financial burden of care and support services to PLHA and households in South India.. AIDS Care.

[29] WHO (2009). World Health Statistics.

[30] Russell S (1996). Ability to pay for health care: concepts and evidence.. Health Policy Plan.

[31] Pradhan M, Prescott N (2002). Social risk management options for medical care in Indonesia.. Health Econ.

[32] Van Damme W, Meessen B, Por I, Kober K (2003). Catastrophic health expenditure.. Lancet.

[33] Flores G, Krishnakumar J, O'Donnell O, van Doorslaer E (2008). Coping with health-care costs: implications for the measurement of catastrophic expenditures and poverty.. Health Econ.

[34] International Monetary Fund (IMF) (2009). Côte d'Ivoire: Poverty Reduction Strategy Paper.

[35] Lu C, Chin B, Li G, Murray CJ (2009). Limitations of methods for measuring out-of-pocket and catastrophic private health expenditures.. Bull World Health Organ.

[36] Russel S, Gilson L (2006). Are health services protecting the livelihoods the urban poor in Sri Lanka? Findings from two low-income areas of Colombo.. Soc Sci Med.

[37] Souteyrand YP, Collard V, Moatti JP, Grubb I, Guerma T (2008). Free care at the point of service delivery: a key component for reaching universal access to HIV/AIDS treatment in developing countries.. AIDS.

[38] Zachariah R, Van Engelgem I, Massaquoi M, Kocholla L, Manzi M (2008). Payment for antiretroviral drugs is associated with a higher rate of patients lost to follow-up than those offered free-of-charge therapy in Nairobi, Kenya.. Trans R Soc Trop Med Hyg.

[39] Mukherjee JS, Ivers L, Leandre F, Farmer P, Behforouz H (2006). Antiretroviral therapy in resource-poor settings. Decreasing barriers to access and promoting adherence.. J Acquir Immune Defic Syndr.

[40] Vinard P, Diop K, Taverne B, Coriat B (2008). Implementing funding modalities for free access: The case for a “purchasing fund system” to cover medical care.. The political Economy of HIV/AIDS in Developing Countries TRIPS, Public Health Systems and Free Access.

[41] Palmer N, Mueller DH, Gilson L, Mills A, Haines A (2004). Health financing to promote access in low income settings-how much do we know?. Lancet.

[42] James CD, Hanson K, McPake B, Balabanova D, Gwatkin D (2006). To retain or remove user fees?: reflections on the current debate in low- and middle-income countries.. Appl Health Econ Health Policy.

[43] Gilson L, Kalyalya D, Kuchler F, Lake S, Oranga H (2001). Strategies for promoting equity: experience with community financing in three African countries.. Health Policy.

[44] Ranson MK (2002). Reduction of catastrophic health care expenditures by a community-based health insurance scheme in Gujarat, India: current experiences and challenges.. Bull World Health Organ.

[45] Bennett S (2004). The role of community-based health insurance within the health care financing system: a framework for analysis.. Health Policy Plan.

[46] Carrin G, Waelkens MP, Criel B (2005). Community-based health insurance in developing countries: a study of its contribution to the performance of health financing systems.. Trop Med Int Health.

[47] Molyneux C, Hutchison B, Chuma J, Gilson L (2007). The role of community-based organizations in household ability to pay for health care in Kilifi District, Kenya.. Health Policy Plan.

[48] Leive A, Xu K (2008). Coping with out-of-pocket health payments: empirical evidence from 15 African countries.. Bull World Health Organ.

[49] Carrin G, Mathauer I, Xu K, Evans DB (2008). Universal coverage of health services: tailoring its implementation.. Bull World Health Organ.

[50] Lange JM, Schellekens OP, Lindner M, van der Gaag J (2008). Public-private partnerships and new models of healthcare access.. Curr Opin HIV AIDS.

[51] Samb B, Evans T, Dybul M, Atun R, Moatti JP (2009). An assessment of interactions between global health initiatives and country health systems.. Lancet.

